# Influence of psychpathic personality traits on anxiety in a mixed reality Study

**DOI:** 10.1192/j.eurpsy.2023.1133

**Published:** 2023-07-19

**Authors:** J. Fuss, A. Voulgaris

**Affiliations:** 1 Institute for Sex Research and Forensic Psychiatry, Universitätsklinikum Essen, Essen; 2 Institute for Sexual Medicine and Forensic Psychiatry, Universitätsklinikum Hamburg Eppendorf, Hamburg, Germany

## Abstract

**Introduction:**

The personality construct of psychopathy consists of severe deficits in behavior, emotion and cognition, often categorized in the two dimensions affective-interpersonal and antisocial-lifestyle. Research indicates that a lack of anxiety and fear play an important role in psychopathic personalities. Understanding the interplay of psychopathic traits, fear and threat processing and reactive behavior is important due to its implications for risky and potentially antisocial behavior.

**Objectives:**

We conducted a mixed reality study using the elevated plus-maze in a non-clinical sample to test anxiety-related behavior in correlation to psychopathic personality traits. Our hypothesis was that higher psychopathy would lead to higher expression of risky behavior and, thus, to longer time on open arms, higher number of entries to open arms and reduced latency for a first visit on open arms and for open arm endexploration.

**Methods:**

Healthy volunteers were recruited (N=170) and completed the Sensation Seeking Scale V (SSSV), the Acrophobia Questionnaire (AQ), and the Brief Questionnaire of Psychopathic Personality Traits. The included subjects were tested on the human elevated plus-maze, which consists of a physical life-sized wooden platform and its representation in a virtual environment. Data recording was performed via the virtual reality tracking system (HTC Vive Base Station®, Seattle, USA) and custom soft-ware (A+ cross®) using the following parameters: total time spent on open arms (time on open arms), number of entries to open arms, latency for the first entry of an open arm (latency 1st visit) and time until subjects reach the end of an open arm (latency endexploration).

Statistical analyses were carried out using IBM SPSS Statistics 23.0 (IBM Corp., Armonk, NY, USA) and R software (R version 4.0.2). Pearson correlation was performed to assess associations between psychopathic traits and measures of anxiety-related constructs.

**Results:**

Reduced levels of anxiety were associated with higher psychopathic traits. This was reflected in a correlation between the PPT sum score and all measures of anxiety-like behavior on the EPM (time on open arms: R = .29, p < 0.001; number of entries to open arms: R = .32, p < 0.001; latency 1st visit: R = -.29, p < 0.001; latency endexploration: R = -.30, p < 0.001). Psychopathic traits were also negatively correlated with subjective levels of anxiety on the EPM (R = -.23, p = 0.004). Sensation seeking (SSSV) (R =.33, p < 0.001) but not general levels of acrophobia (ACQ) (R = -.13, p = 0.11) were moreover associated with psychopathic personality traits.

**Conclusions:**

In light of the ongoing discussion, our results demonstrate a correlation between psychopathic personality traits and anxiety-related behavior in a non-clinical sample. This supports the theory of a lack of fear in psychopathy and may influence risky and potentially harmful behavior in this population.

**Disclosure of Interest:**

None Declared

